# Reply to: Comments on: Differential IgG4-Producing Plasma Cell Infiltration in Non- and Post-Transplant Plasma Cell Hepatitis

**DOI:** 10.3389/ti.2022.10679

**Published:** 2022-08-05

**Authors:** Brian Harris Horwich, Jeffrey A. Kahn, Takeshi Saito

**Affiliations:** Keck School of Medicine, University of Southern California, Los Angeles, CA, United States

**Keywords:** IgG4, rejection, alloimmunity, autoimmunity, plasma cell hepatitis

We would like to thank Drs. Aguilera and Sousa for their thoughtful commentary on our recent publication “*Differential IgG4-Producing Plasma Cell Infiltration in Non- and Post-Transplant Plasma Cell Hepatitis*.” In particular, we would like to thank the authors for calling their important work to our attention.

Their identification of Glutathione S-transferase T1 (GSTT1) gene mismatch between donor and recipient, specifically Donor (−)/Recipient (+), as a potential predictor for developing plasma cell-rich rejection (PCR) was a critical breakthrough in this field. Additionally, their work also suggests that serologic evaluation of anti-GSTT1 antibodies may be a useful marker in PCR diagnosis and disease response to corticosteroid therapy (1). It is satisfying that the findings from our histopathologic assessment of post-transplant plasma cell hepatitis correlates with their observations (2).

As the authors’ mentioned, the findings of our study and their prior work may be of particular clinical relevance in the evaluation of patients for whom a pre-liver transplantation (LT) diagnosis was not established (i.e., those in fulminant liver failure of unknown etiology). The current diagnostic algorithm does not provide adequate guidance with unclear pre-LT diagnosis, reflecting the fact that it is entirely clinical context-based, but not immuno-pathobiology-based diagnosis (3). Accordingly, the roles of Immunoglobulin subclass 4 (IgG4) immunostaining and serologic antibody testing are not well-established in differentiating PCR from recurrent autoimmune hepatitis (rAIH). This is despite prior literature demonstrating that PCR is not a immunologically homogenous entity by its current definition (4).

Consequently, our studies suggest a potential complementary approach to the evaluation of these individuals. One possible diagnostic algorithm could be evaluating the IgG4 Positivity by immunohistochemistry in combination with anti-GSTT1 antibody serologic testing. Based on the findings of our studies, high IgG4 Positivity and elevated anti-GSTT1 antibodies would be highly suggestive of PCR. Conversely, low IgG4 Positivity and absent anti-GSTT1 antibodies may be confer a diagnosis of rAIH. A study in which IgG4 Positivity and anti-GSTT1 antibodies are identified in the same subjects is warranted. Additionally, as our works only evaluated a combined 28 cases of PCR and rAIH, further studies with a larger cohort are needed (1, 2).

## Abbreviations

IgG4, immunoglobulin subclass 4; GSTT1, Glutathione S-transferase T1; LT, liver transplantation; PCR, plasma cell-rich rejection; rAIH, recurrent autoimmune hepatitis.
